# Scopolamine effects on functional brain connectivity: a pharmacological model of Alzheimer’s disease

**DOI:** 10.1038/srep09748

**Published:** 2015-07-01

**Authors:** R. Bajo, S. Pusil, M. E. López, L. Canuet, E. Pereda, D. Osipova, F. Maestú, E. Pekkonen

**Affiliations:** 1 Laboratory of Cognitive and Computational Neuroscience (UCM-UPM). Centre for Biomedical Technology (CTB), Madrid, Spain; 2 Department of Industrial Engineering & Institute for Biomedical Technology (ITB). University of La Laguna, Spain; 3 BioMag Laboratory, Engineering Centre, Helsinki University Central Hospital, Helsinki, Finland; 4 Department of Neurology, University of Helsinki, Helsinki, Finland

## Abstract

Scopolamine administration may be considered as a psychopharmacological model of Alzheimer’s disease (AD). Here, we studied a group of healthy elderly under scopolamine to test whether it elicits similar changes in brain connectivity as those observed in AD, thereby verifying a possible model of AD impairment. We did it by testing healthy elderly subjects in two experimental conditions: glycopyrrolate (placebo) and scopolamine administration. We then analyzed magnetoencephalographic (MEG) data corresponding to both conditions in resting-state with eyes closed. This analysis was performed in source space by combining a nonlinear frequency band-specific measure of functional connectivity (phase locking value, PLV) with network analysis methods. Under scopolamine, functional connectivity between several brain areas was significantly reduced as compared to placebo, in most frequency bands analyzed. Besides, regarding the two complex network indices studied (clustering and shortest path length), clustering significantly decreased in the alpha band while shortest path length significantly increased also in alpha band both after scopolamine administration. Overall our findings indicate that both PLV and graph analysis are suitable tools to measure brain connectivity changes induced by scopolamine, which causes alterations in brain connectivity apparently similar to those reported in AD.

Functional Connectivity (FC) has been proposed^1^,^2^,^3^ as an approach to describe how brain regions coordinate to support higher cognitive functions. FC measures the statistical interdependencies between two time series (brain signals) and is related to the ability to communicate information between brain regions which underlies many cognitive functions. Since FC provides information on the relationship between simultaneously recorded signals^4^, it has been extensively used to gain insight into the interactions between the corresponding brain regions. Subsequently, with the aim to describe the functional brain network associated to a FC pattern, different graph theoretic methods have been used^3^,^5^,^6^,^7^. Graph theory allows representing a complex set of relationships (i.e. a network) as an ensemble of nodes interconnected by links. Here, the nodes are the MEG sensors with their corresponding electrophysiological information, and the links between two MEG sensors are the corresponding estimation of the strength of FC, in our case, the Phase Locking Value (PLV), as detailed below.

Scopolamine, a muscarinic receptor antagonist, produces a blocking of the activity of the muscarinic acetylcholine receptor, and the concomitant appearance of transient cognitive amnesia and electrophysiological changes, which resemble those observed in Alzheimer’s Disease (AD)^8^,^9^. Indeed, to date, several studies have explored neurophysiological changes associated with scopolamine injection mirroring those observed in AD^10^. After scopolamine administration, quantitative electroencephalogram resting state studies have found decreased power in alpha and beta bands, and increased delta and theta activity^11^,^12^,^13^,^14^. Additionally, studies using coherence during resting-state have shown a decrease in this measure after scopolamine^12^,^15^. Further, an interesting recent study on reconstructed EEG sources using LORETA^9^, found changes in brain activity following scopolamine administration, mainly at the precuneus. Taken together, these neurophysiological and cognitive alterations suggest that the administration of scopolamine may be an adequate approximation to simulate the modifications in brain activity that take place in AD^13^.

Regarding the application of FC to electrophysiological data in the study of AD, several studies have showed an increase in slow band (i.e. delta band) connectivity in AD and a reduction in FC among different cortical areas (e.g. fronto-parietal, fronto-temporal and parieto-occipital circuits) in alpha and beta bands^5^,^16^,^17^,^18^. Additionally, Osipova *et al.*^12^ studied the effect of scopolamine administration on MEG spectral power and coherence. They measured spectral power and coherence over 7 brain regions (frontal, central, left temporal, right temporal, left parietal, right parietal, and occipital) in 3 frequency bands (theta: 4-8 Hz, alpha: 8-13 Hz and beta: 13-22 Hz). In this work, however, coherence was computed in the sensor space between only 8 MEG channels, which limited the implication of the results. Instead, we re-analyzed here the whole data set (using all MEG channels available: 122) by combining graph theory with a nonlinear index of phase synchronization, the PLV, to study FC in the source space, between 88 brain regions, in 5 frequency bands. To the best of our knowledge, no research hitherto has evaluated the effects of scopolamine on the network MEG structure at the source domain. We hypothesize that functional MEG connectivity in source space will decrease after scopolamine administration as compared to placebo, and also that the architecture of the functional brain network, assessed by graph theory analysis, will resemble that observed in AD´s patients. Besides, the current study will also provide evidences of a model of AD and of the usefulness of FC analysis in the study and description of brain networks.

## Materials and Methods

The subjects of the present study are a subset of those from Osipova *et al.*^12^, who were carefully selected according to the quality of the recordings. They consisted of 7 healthy right-handed elderly volunteers (59-80 years and 6 females), recruited from a community cultural center for elderly citizens. Scopolamine hydrobromide (0.3 mg) or glycopyrrolate (0.2 mg), which acted as placebo without penetrating the blood-brain barrier, was given intravenously to each subject in a double blind, cross-over design. The subjects were instructed to move as little as possible and remain awake, while keeping their eyes closed. Magnetic fields were then recorded during this resting state using a 122-channel whole-head MEG device (Neuromag Ltd, Finland) confined in a magnetically shielded room. Raw data were sampled at 400 Hz and band-pass filtered between 0.03–150 Hz. Time-segments containing artifacts (e.g. amplitudes over 3 pT/cm), were considered extra-cerebral noise. Analyses were performed for the commonly used frequency bands, in order to facilitate comparison with other studies: delta (0.5–4 Hz), theta (4–8 Hz), alpha (8–12 Hz), beta (13–30 Hz) and gamma (30–45 Hz).

To reconstruct the neural MEG sources, we selected 88 brain regions derived from a BrainVisa atlas^19^, using Brainstorm software^20^ to define anatomical regions of interest. This number of regions was enough to describe the functional brain network, considering the available 122 MEG channels. Besides an expert neurologist certified its suitability from the point of view of brain functionality. Because brain MRIs of the subjects were not available, we calculated source activity by using the default anatomy in this toolbox, which consisted of the segmented cortical surface (15000 vertices) of the MNI/Colin27 brain^21^. We chose overlapping sphere method (OS) with Brainstorm default parameter settings to solve the forward problem. This approach in combination with minimum-norm method (MNM) provides the better outcome when the MRIs are not available^22^. Next, a noise covariance matrix was calculated to estimate noise level in the MEG recordings. Then, weighted minimum-norm estimation wMNE^23^ was chosen to compute source activity. The grid of sources (dipoles) was defined by the cortex surface template of the default anatomy; each vertex of this surface was considered as a dipole. Finally, we extracted the time series from these cortical ROIs as the average of all dipole’s signals within each area of the atlas mentioned above.

The abovementioned PLV was used to measure functional connectivity between all 88 brain regions, in each frequency band. First, data were band-pass filtered using a zero-phase distortion finite impulse response filter with a bandwidth of 2 Hz. Then, we obtain the complex analytic signal from each of the brain regions by using the Hilbert transform (see, e.g.,^4^ for details). Subsequently, the PLV between the time series *x(k)* and *y(k)* from two brain regions is defined^24^ as:
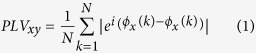
where *N* is the length of the time series (in our case, *N*=96000, which equals 120s.) and φ_*x*_*(k)* (resp. φ_*y*_*(k))* is the phase of *x(k)* (resp. *y(k)*). Finally, the PLV in each of the frequency bands (delta, theta…) was obtained by averaging the corresponding PLVs in the given frequency range.

Additionally, we described the architecture of the functional brain network of the subjects by means of two indices: the clustering coefficient (*C*) and the average shortest path length (*L*). The former one is a measure of graph segregation that indicates how the neighborhoods of a node are interconnected. The latter one is an estimation of network integration, which measures the average of all the shortest distances between pairs of nodes in the network^25^,^26^,^27^. We calculated the mentioned network parameters (C and L) from the weighted matrix (PLV values) using the “Brain Connectivity Toolbox”^25^ (http://www.brain-connectivity-toolbox.net). The size of the graph was 88 nodes (corresponding with the 88 source space regions described). Indices are un-normalized.

Statistical differences between experimental conditions (scopolamine vs. placebo), were investigated for both *C* and *L* by means of a paired t-test at the p<0.05 level. In the case of the PLV, we first applied a Kruskal-Wallis test and further a non-parametric permutation test with 1000 permutations to each of the significantly different PLVs to correct for multiple comparisons across the full range of functional connectivity pairings. In this latter case, we set the level of significance at p<0.005.

Ethics statement: Methods were carried out in “accordance” with the approved guidelines. The study was approved by the National Agency for Medicine of Finland and the Ethics Committee of the local University Hospital (Helsinki University Central Hospital, Helsinki). Besides, a written informed consent was obtained from all the subjects after a detailed explanation of the procedures.

## Results

**Figure 1:**
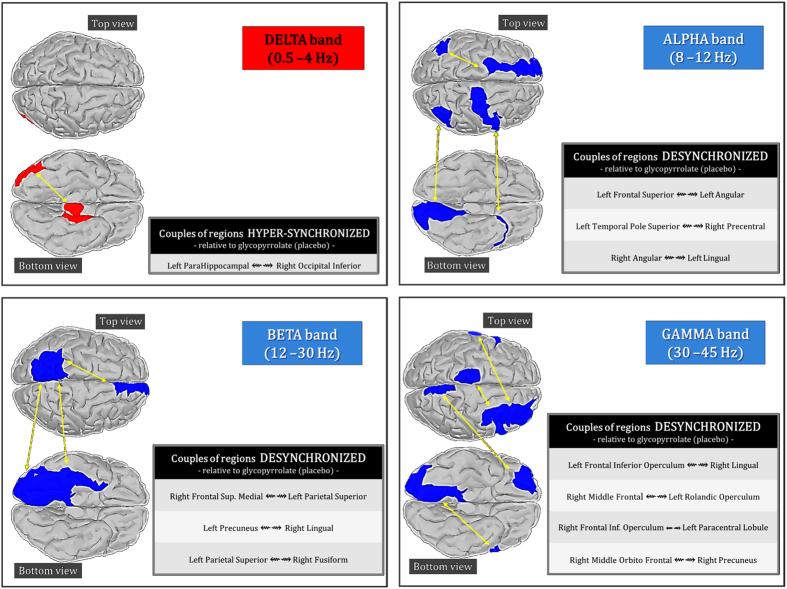
Significant differences in PLV between the Glycopyrrolate (placebo) and the scopolamine conditions for different frequency bands (at the p<0.005 level). In the delta band (top left) the synchronization between left parahippocampal and right occipital interior cortex increases under the scopolamine. On the contrary in alpha (top right), beta (bottom left) and gamma bands (bottom right) the PLV decreases between different brain regions under scopolamine.

Scopolamine significantly increased connectivity in the delta band and significantly reduced it in the alpha, beta and gamma band as compared to glycopyrrolate (placebo). Fig. 1 shows the distribution of these changes across different cortical regions. Particularly, in the delta band scopolamine increased connectivity between the left parahippocampal and the right inferior occipital cortex. Additionally, we found reduced connectivity in the alpha band between the left superior frontal and the left angular cortex, the left superior temporal pole and the right precentral cortex, and between the right angular and the left lingual cortex. In the beta band, we found significantly reduced connectivity, specifically between the left medial superior frontal and the left superior parietal cortex, the left precuneus and the right lingual cortex, and between the left superior parietal and right fusiform cortex. Finally, in the gamma band, there was significantly reduced connectivity between the right middle frontal cortex and the left rolandic operculum, and between the right middle orbito-frontal cortex and the right precuneus. We also found reduced gamma band connectivity of the left inferior frontal operculum with the right lingual and left paracentral lobule.

**Figure 2:**
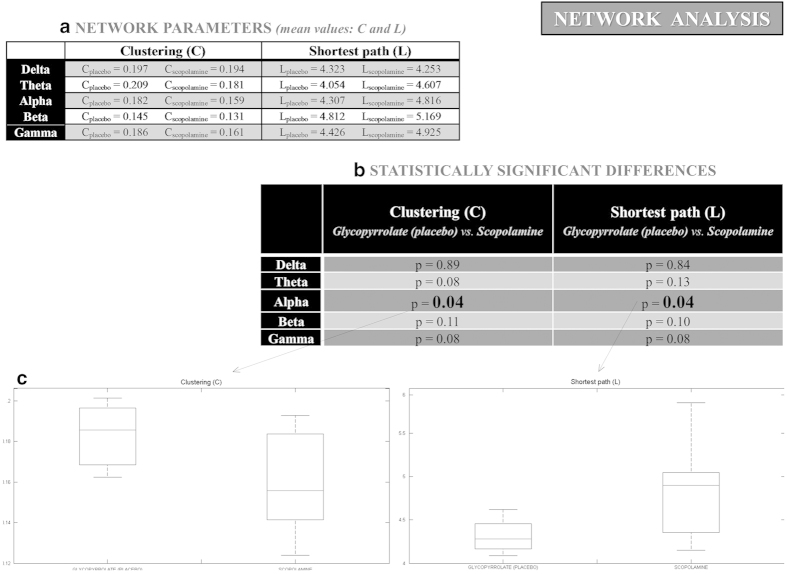
(**a**) Network parameters (C and L mean values) for each frequency band and condition (placebo and Scopolamine); (b) Statistically significant differences (p values) for C and L, when comparing both conditions (Glycopyrrolate (placebo) vs. Scopolamine) for each frequency band; (**c**) In alpha band, C shows a significant reduction under scopolamine as compared to placebo, while L shows a significant increased.

As for the brain network indices, C shows a significant reduction (p<0.04) and L a significant increased (p<0.04) both of them under scopolamine as compared to placebo in the alpha band, as depicted in Fig. 2.

## Discussion

We have investigated here whether functional brain networks were impaired after the administration of a cholinergic receptor antagonist (scopolamine) in healthy elderly subjects. Additionally we evaluated whether the effects of scopolamine on FC and functional MEG network structure mirrored the disconnection syndrome reported in AD patients^5^,^28^,^29^. Our findings showed that, compared to placebo, scopolamine significantly increases MEG phase synchronization in the delta band, specifically between the left medial temporal region (i.e. para-hippocampus) and the contralateral occipital cortex (taking into account the reservations, in terms of precision, which involves the calculation of such deep sources). This increment in slow band connectivity may reflect the excitotoxicity or pathological effect of scopolamine administration and was probably the result of the cholinergic cortical deafferentation from subcortical structures^16^,^17^,^18^. Scopolamine, however, decreased functional connectivity between several cortical regions in alpha, beta and gamma bands. In the alpha band, this reduction involved the left fronto-parietal and the inter-hemispheric fronto-temporal and parieto-occipital connectivity. Abnormalities in faster frequencies were characterized by reduced inter-hemispheric connections of the left parietal cortex (i.e. beta band) and the frontal lobes (i.e. gamma band). Using another index of phase synchronization, the so-called phase lag index (PLI), Stam *et al.*^5^ reported that MEG resting-state FC was decreased in AD patients for frequencies above 8 Hz. In fact, and consistent with our results, these authors found lower left fronto-parietal connectivity values in AD relative to healthy controls for the alpha band, as well as a disruption in fronto-temporal and parieto-occipital circuits. There was also a significant impairment of right parietal lobe connections. For faster frequencies (13-30 Hz), they also observed a predominant decrease in interhemispheric frontal connectivity and in fronto-parietal circuits. Moreover, consistent with our outcomes, most studies hitherto have found decreased functional connectivity mainly in alpha and beta bands in AD patients compared to healthy controls^10^,^16^,^30^,^31^,^32^.

Several cortical regions showing scopolamine-induced FC decrease in this study (e.g. precuneus, medial frontal and inferior parietal cortex) are part of the default mode network (DMN), which is known to be active during resting and attenuated during cognitive tasks. Specific regions of the DMN, including the precuneus in the medial parietal cortex, are selectively vulnerable to early pathological changes in AD^33^. Besides, precuneus was identified as a critical anatomical area^9^,^34^,^35^, thus suggesting that this structure could be a “network hub” which is affecting other functional regions. Beyond the DMN, other cortical areas showed loss of FC. For instance, we also found abnormal connectivity in areas of the anterior temporal network, including the hippocampus and the temporal pole^36^. Interestingly, this network is involved in memory function, which is typically impaired in AD^33^.

It is noteworthy that cortical areas that belong to other brain networks also exhibited disruption in FC after scopolamine administration. These networks include the fronto-parietal and the dorso-lateral prefrontal ones, which are involved in attention and executive functions, respectively. As mentioned above, Iturria-Medina *et al.*^35^ identified “the most indispensable and critical anatomical areas in the brain network”, which among others also included: superior parietals and superior frontals. These regions that were also disrupted by scopolamine administration are important brain network hubs. Besides, this finding is in line with that of a recent resting-state fMRI study^37^ reporting disrupted functional connectivity between multiple brain networks in AD. Taken together, these findings suggest that there is scopolamine-related disorganization of several cortical networks, where the DMN appears to be particularly compromised.

In addition to connectivity analysis in source space, we examined changes in the large-scale structure of resting-state functional brain networks using concepts from graph theory. In a recent review, Tijms *et al.*^29^ claim that the un-normalized path length (L) was most consistently reported (6 of 8 studies) to be increased in AD. This increase has been usually interpreted to result from the loss of connectivity. Moreover, regarding the clustering (C) index, these authors indicate that no definite differences have been found between both groups (AD and Controls), since the studies reviewed showed considerable variability. Hence, the present study is consistent with the findings reported the literature about the increase of path length in AD, and supports the idea that the clustering index decreases in the lower alpha band in AD patients as compared to healthy subjects^5^.

Thus, what we have found here supports the idea that scopolamine may be considered as a psychopharmacological model of AD, since both the drug and the disease produce a similar disruption in FC and brain network architecture. Importantly, donepezil, a drug belonging to the family of cholinesterase inhibitors, has been reported to improve abnormalities in FC of the hippocampus and prefrontal areas^38^, which provides further support to this argument.

Overall, the present study shows that the pharmacological induction of amnesia in a group of healthy elderly subjects seems to produce a similar disruption of FC and functional MEG brain networks organization to that reported in AD patients. At any rate, some reservations must be pointed out before confirming that scopolamine administration produces similar alterations that those observed in AD patients. It is necessary to indicate that in the present study we did not perform any correlations between FC and the behavioral data while subjects were under scopolamine condition. In addition, we did not analyze an AD cohort but rather discuss our findings in the context of the existing literature of AD effects on functional MEG connectivity^5^,^10^,^18^,^39^. From these results, and considering the limitations discussed, we conclude that the assessment of functional MEG connectivity and the subsequent characterization of the brain network structure may be a useful approach to understand AD, and provide new strong evidences of the usefulness of combining both methodologies to study brain function from multivariate neuroimaging data in health and disease.

## Additional Information

**How to cite this article**: Bajo, R. *et al.* Scopolamine effects on functional brain connectivity: a pharmacological model of Alzheimer's disease. *Sci. Rep.* **5**, 9748; doi: 10.1038/srep09748 (2015).

## References

[b1] Friston K. J. Functional and effective connectivity in neuroimaging: a synthesis. Hum. Brain Mapp. 2, 56–78 (1994).

[b2] Friston K. J. Brain function, nonlinear coupling, and neuronal transients. Neuroscientist 7, 406–418 (2001). 11597100 10.1177/107385840100700510

[b3] Sporns O., Chialvo D. R., Kaiser M. & Hilgetag C. C. Organization, development and function of complex brain networks. Trends Cogn. Sci. 8, 418–25 (2004). 15350243 10.1016/j.tics.2004.07.008

[b4] Pereda E., Quiroga R. Q. & Bhattacharya J. Nonlinear multivariate analysis of neurophysiological signals. Prog. Neurobiol. 77, 1–37 (2005). 16289760 10.1016/j.pneurobio.2005.10.003

[b5] Stam C. J. *et al.* Graph theoretical analysis of magnetoencephalographic functional connectivity in Alzheimer’s disease. Brain 132, 213–224 (2009). 18952674 10.1093/brain/awn262

[b6] Buldú J. M. *et al.* Reorganization of functional networks in mild cognitive impairment. PLoS One 6, e19584DOI: 10.1371/journal.pone.0019584 (2011). 21625430 PMC3100302

[b7] Achard S. & Bullmore E. Efficiency and cost of economical brain functional networks. PLoS Comput. Biol. 3, 0174–0183 (2007). 10.1371/journal.pcbi.0030017 PMC1794324 17274684

[b8] Spinks A. & Wasiak J. Scopolamine (hyoscine) for preventing and treating motion sickness. Cochrane database Syst. Rev. DOI: 10.1002/14651858 (2011). PMC7138049 21678338

[b9] Reis P. M. R., Eckhardt H., Denise P., Bodem F. & Lochmann M. Localization of scopolamine induced electrocortical brain activity changes, in healthy humans at rest. J. Clin. Pharmacol. 53, 619–625 (2013). 23650014 10.1002/jcph.83

[b10] Jeong J. EEG dynamics in patients with Alzheimer’s disease. Clin. Neurophysiol. 115, 1490–1505 (2004). 15203050 10.1016/j.clinph.2004.01.001

[b11] Sannita W. G., Maggi L. & Rosadini G. Effects of scopolamine (0.25-0.75 mg i.m.) on the quantitative EEG and the Neuropsychological status of healthy volunteers. Pharmacoelectroencephalography. 17, 199–205 DOI: 10.1159/000118365 (1987). 3441275

[b12] Osipova D. *et al.* Effects of scopolamine on MEG spectral power and coherence in elderly subjects. Clin. Neurophysiol. 114, 1902–1907 (2003). 14499752 10.1016/s1388-2457(03)00165-2

[b13] Ebert U. & Kirch W. Scopolamine model of dementia: electroencephalogram findings and cognitive performance. Eur. J. Clin. Invest. 28, 944–949 (1998). 9824440 10.1046/j.1365-2362.1998.00393.x

[b14] Ebert U., Grossmann M., Oertel R., Gramatté T. & Kirch W. *Pharmacokinetic-pharmacodynamic modeling of the electroencephalogram effects of scopolamine in healthy volunteers*. J. Clin. Pharmacol. 41, 51–60 (2001). 11144994 10.1177/00912700122009836

[b15] Sloan E. P., Fenton G. W. & Standage K. P. Anticholinergic drug effects on quantitative electroencephalogram, visual evoked potential, and verbal memory. Biol. Psychiatry 31, 600–606 (1992). 1581439 10.1016/0006-3223(92)90246-v

[b16] Locatelli T., Cursi M., Liberati D., Franceschi M. & Comi G. EEG coherence in Alzheimer’s disease. Electroencephalogr. Clin. Neurophysiol. 106, 229–237 (1998). 9743281 10.1016/s0013-4694(97)00129-6

[b17] Pijnenburg Y. A. L. *et al.* EEG synchronization likelihood in mild cognitive impairment and Alzheimer’s disease during a working memory task. Clin. Neurophysiol. 115, 1332–1339 (2004). 15134700 10.1016/j.clinph.2003.12.029

[b18] Stam C. J. *et al.* Magnetoencephalographic evaluation of resting-state functional connectivity in Alzheimer’s disease. Neuroimage 32, 1335–1344 (2006). 16815039 10.1016/j.neuroimage.2006.05.033

[b19] Tzourio-Mazoyer N. *et al.* Automated anatomical labeling of activations in SPM using a macroscopic anatomical parcellation of the MNI MRI single-subject brain. Neuroimage 15, 273–89 (2002). 11771995 10.1006/nimg.2001.0978

[b20] Tadel F., Baillet S., Mosher J. C., Pantazis D. & Leahy R. M. Brainstorm: a user-friendly application for MEG/EEG analysis. *Comput. Intell. Neurosci*. DOI: 10.1155/2011/879716 (2011). PMC3090754 21584256

[b21] Holmes C. J. *et al.* Enhancement of MR images using registration for signal averaging. J. Comput. Assist. Tomogr. 22, 324–33 (1998). 9530404 10.1097/00004728-199803000-00032

[b22] Henson R. N., Mattout J., Phillips C. & Friston K. J. Selecting forward models for MEG source-reconstruction using model-evidence. Neuroimage 46, 168–176 (2009). 19457358 10.1016/j.neuroimage.2009.01.062 PMC2912517

[b23] Mosher J. C., Baillet S. & Leahy R. M. Equivalence of linear approaches in bioelectromagnetic inverse solutions. in IEEE Work. Stat. Signal Process. 2003 294–297 IEEE, 2003). DOI: 10.1109/SSP.2003.1289402 (2003).

[b24] Mormann F., Lehnertz K., David P. & Elger E., C. Mean phase coherence as a measure for phase synchronization and its application to the EEG of epilepsy patients. Phys. D Nonlinear Phenom. 144, 358–369 (2000).

[b25] Rubinov M. & Sporns O. Complex network measures of brain connectivity: uses and interpretations. Neuroimage 52, 1059–1069 (2010). 19819337 10.1016/j.neuroimage.2009.10.003

[b26] Sporns O. Network attributes for segregation and integration in the human brain. Curr. Opin. Neurobiol. 23, 162–171 (2013). 23294553 10.1016/j.conb.2012.11.015

[b27] Sporns O. Contributions and challenges for network models in cognitive neuroscience. Nat. Neurosci. 17, 652–60 (2014). 24686784 10.1038/nn.3690

[b28] Bajo R. *et al.* Functional connectivity in mild cognitive impairment during a memory task: implications for the disconnection hypothesis. J. Alzheimers. Dis. 22, 183–193 (2010). 20847450 10.3233/JAD-2010-100177

[b29] Stam C. J., Y, V. D. M., Yal P. & Eeg S. P. EEG synchronization in mild cognitive impairment and Alzheimer’s disease. Acta Neurol. Scand. 108, 90–96 (2003). 12859284 10.1034/j.1600-0404.2003.02067.x

[b30] Babiloni C. *et al.* Mapping distributed sources of cortical rhythms in mild Alzheimer’s disease. A multicentric EEG study. Neuroimage 22, 57–67 (2004). 15109997 10.1016/j.neuroimage.2003.09.028

[b31] Koenig T. *et al.* Decreased EEG synchronization in Alzheimer’s disease and mild cognitive impairment. Neurobiol. Aging 26, 165–171 (2005). 15582746 10.1016/j.neurobiolaging.2004.03.008

[b32] Sperling R. A. *et al.* Functional alterations in memory networks in early Alzheimer’s disease. Neuromolecular Med. 12, 27–43 (2010). 20069392 10.1007/s12017-009-8109-7 PMC3036844

[b33] Iturria-Medina Y. *et al.* Characterizing brain anatomical connections using diffusion weighted MRI and graph theory. Neuroimage 36, 645–660 (2007). 17466539 10.1016/j.neuroimage.2007.02.012

[b34] Iturria-Medina Y., Sotero R. C., Canales-Rodríguez E. J., Alemán-Gómez Y. & Melie-García L. Studying the human brain anatomical network via diffusion-weighted MRI and Graph Theory. Neuroimage 40, 1064–1076 (2008). 18272400 10.1016/j.neuroimage.2007.10.060

[b35] Wink A. M., Bernard F., Salvador R., Bullmore E. & Suckling J. Age and cholinergic effects on hemodynamics and functional coherence of human hippocampus. Neurobiol. Aging 27, 1395–1404 (2006). 16202481 10.1016/j.neurobiolaging.2005.08.011

[b36] Brier M. R. *et al.* Loss of intranetwork and internetwork resting state functional connections with Alzheimer’s disease progression. J. Neurosci. 32, 8890–8899 (2012). 22745490 10.1523/JNEUROSCI.5698-11.2012 PMC3458508

[b37] Watts D. & Strogatz S. Collective dynamics of “small-world” networks. Nature 393, 440–442 (1998). 9623998 10.1038/30918

[b38] Zaidel L. *et al.* Donepezil effects on hippocampal and prefrontal functional connectivity in Alzheimer’s disease: preliminary report. J. Alzheimers. Dis. 31 Suppl 3, S221–6 (2012). 22886013 10.3233/JAD-2012-120709 PMC3749074

[b39] Berendse H. W., Verbunt J. P., Scheltens P., van Dijk B. W. & Jonkman E. J. Magnetoencephalographic analysis of cortical activity in Alzheimer’s disease: a pilot study. Clin. Neurophysiol. 111, 604–612 (2000). 10727911 10.1016/s1388-2457(99)00309-0

